# DNA microarray-based assessment of virulence potential of Shiga toxin gene-carrying *Escherichia coli* O104:H7 isolated from feedlot cattle feces

**DOI:** 10.1371/journal.pone.0196490

**Published:** 2018-04-30

**Authors:** Pragathi B. Shridhar, Isha R. Patel, Jayanthi Gangiredla, Lance W. Noll, Xiaorong Shi, Jianfa Bai, Christopher A. Elkins, Nancy Strockbine, T. G. Nagaraja

**Affiliations:** 1 Department of Diagnostic Medicine and Pathobiology; 2 Division of Molecular Biology, Center for Food Safety and Applied Nutrition, United States Food and Drug Administration, Laurel, Maryland, United States of America; 3 Veterinary Diagnostic Laboratory Kansas State University, Manhattan, Kansas, United States of America; 4 Division of Foodborne, Waterborne, and Environmental Diseases, National Center for Emerging and Zoonotic Infectious Diseases, CDC, Atlanta, Georgia, United States of America; The Pennsylvania State University, UNITED STATES

## Abstract

*Escherichia coli* O104:H4, a hybrid pathotype reported in a large 2011 foodborne outbreak in Germany, has not been detected in cattle feces. However, cattle harbor and shed in the feces other O104 serotypes, particularly O104:H7, which has been associated with sporadic cases of diarrhea in humans. The objective of our study was to assess the virulence potential of Shiga toxin-producing *E*. *coli* (STEC) O104:H7 isolated from feces of feedlot cattle using DNA microarray. Six strains of STEC O104:H7 isolated from cattle feces were analyzed using FDA-*E*. *coli* Identification (ECID) DNA microarray to determine their virulence profiles and compare them to the human strains (clinical) of O104:H7, STEC O104:H4 (German outbreak strain), and O104:H21 (milk-associated Montana outbreak strain). Scatter plots were generated from the array data to visualize the gene-level differences between bovine and human O104 strains, and Pearson correlation coefficients (r) were determined. Splits tree was generated to analyze relatedness between the strains. All O104:H7 strains, both bovine and human, similar to O104:H4 and O104:H21 outbreak strains were negative for intimin (*eae*). The bovine strains were positive for Shiga toxin 1 subtype c (*stx*1c), enterohemolysin (*ehxA*), tellurite resistance gene (*terD*), IrgA homolog protein (*iha*), type 1 fimbriae (*fimH*), and negative for genes that code for effector proteins of type III secretory system. The six cattle O104 strains were closely related (r = 0.86–0.98) to each other, except for a few differences in phage related and non-annotated genes. One of the human clinical O104:H7 strains (2011C-3665) was more closely related to the bovine O104:H7 strains (r = 0.81–0.85) than the other four human clinical O104:H7 strains (r = 0.75–0.79). Montana outbreak strain (O104:H21) was more closely related to four of the human clinical O104:H7 strains than the bovine O104:H7 strains. None of the bovine *E*. *coli* O104 strains carried genes characteristic of *E*. *coli* O104:H4 German outbreak strain and unlike other human strains were also negative for Shiga toxin 2. Because cattle *E*. *coli* O104:H7 strains possess *stx*1c and genes that code for enterohemolysin and a variety of adhesins, the serotype has the potential to be a diarrheagenic foodborne pathogen in humans.

## Introduction

*Escherichia coli* O104:H4, a hybrid pathotype possessing genes characteristic of enteroaggregative *E*. *coli* (EAEC) and Shiga toxin (Stx)-producing *E*. *coli* (STEC), was responsible for a large foodborne outbreak of hemorrhagic colitis and hemolytic uremic syndrome in Europe, mainly Germany, in 2011. The O104:H4 serotype was also isolated from patients suffering from bloody diarrhea in the Republic of Georgia in 2009 and comparative genomics revealed that they are nearest neighbors to 2011 German outbreak strains. However, there were structural and nucleotide differences in plasmid and prophage profiles and in antimicrobial resistance between German and Georgian strains [1]. Because Stx-negative EAEC O104:H4 had been previously isolated from humans in African countries, it was suggested that the hybrid pathotype has evolved from EAEC O104:H4 by the uptake of Stx-carrying bacteriophage [2–5]. *Escherichia coli* O104:H4 serotype has not been detected in cattle, unlike other major STEC serotypes that cause human illnesses [6–8]. However, cattle harbor O104 serotypes other than H4 in the gut and shed them in the feces [6, 9, 10]. A *stx*2-carrying O104:H21 serotype, possibly of cattle origin, was implicated in an outbreak of hemorrhagic colitis associated with consumption of raw milk in Helena, Montana in 1994 [11]. We have conducted a study with cattle feces collected from a number of feedlots (n = 29) to determine the prevalence of O104 serogroup and reported that serotype O104:H7 was the most commonly isolated *stx*-carrying O104 serotype [9]. The O104:H7 serotype is reported to be associated with sporadic diarrheal cases in humans [12, 13], however, there has been no evidence to suggest that cattle were the source of human infections. Not much is known about the virulence potential of the strains of *E*. *coli* O104:H7 of cattle origin, particularly in relation to human clinical strains of O104:H7. Therefore, the objectives of our study were to analyze the gene content to assess the virulence potential of cattle O104:H7 strains using FDA-ECID DNA microarray and to compare their virulence gene profiles with that of human STEC O104:H7 (clinical strains), O104:H21 (milk-associated Montana outbreak strain) and O104:H4 (German outbreak) strains.

## Materials and methods

Kansas State University Institutional Biosafety Committee and Institutional Animal Care and Use Committee approved this study.

### *E*. *coli* O104 strains

Six strains of STEC O104:H7 (2013-6-659A, 2013-6-672E, 2013-6-685A, 2013-6-48C, 2013-6-122E and 2013-6-148B), isolated from feedlot cattle fecal samples collected at a Midwest slaughter plant [9], were utilized in this study. Additionally, five strains of human O104:H7 (06–3637, 07–3598, 08–4061, 2011C-3665, and 2012C-3400; Centers for Disease Control and Prevention, Atlanta, GA), human STEC clinical strains of O104:H4 (BAA-2326; German outbreak), and O104:H21 (BAA-178; Montana outbreak) were included in the study.

### Microarray assay and data analysis

The strains were subjected to a custom Affymetrix DNA microarray developed by the FDA for identification and characterization of *E*. *coli* [14]. The array was designed using 368 *E*. *coli* and *Shigella* sequence sets to identify 55,918 annotated open reading frames. The array incorporates 41,932 probe sets, which includes 54 closed chromosomes, 47 closed plasmids, and 267 whole genome shotgun sequences. The array can be used to identify 152 O types, 54 H types, 4 *stx*1 subtypes, 8 *stx*2 subtypes, 48 *eae* subtypes, and other virulence genes. The microarray assay was performed according to the protocol described by Patel et al. 2016. Briefly, each strain was grown overnight in Luria broth (Sigma-Aldrich Co., St. Louis, MO) at 37°C. Total DNA was extracted from 1 ml of the culture and fragmented by a brief digestion with RQ1 RNase-Free DNase I. The 3' end of the digested DNA was labelled with biotin, hybridized to FDA-ECID array and incubated for 16 h at 45°C. The presence or absence of each gene was determined using Robust MultiArray Averaging (RMA) method and MAS5.0 algorithm. Robust MultiArray Averaging (RMA) summarized probe set intensity data is provided in S1 Excel File. In addition to bovine and human O104:H7 strains, other O104 strains of different serotypes (H2, H4, H8, H12, H21, H23) from our culture collection were also included in the phylogenetic analysis, performed using FDA-ECID gene differences data from all the probesets. The tree was developed using Neighbor net algorithm using Neighbor joining method (SplitsTree4). Pearson correlation coefficients (r) were determined to compare the bovine O104:H7 and human (O104:H7, O104:H4, O104:H21) strains. Pair wise scatter plots were also generated to visualize the gene level differences between the strains. Hierarchical cluster dendrogram was generated by array probe set differences for 241 strains including the diarrheagenic *E*. *coli* strains from our culture collection representing the broad diversity across the *E*. *coli* species.

## Results

The gene contents of six O104:H7 strains, isolated from cattle feces, were compared to human O104:H7 strains associated with sporadic diarrhea, and German outbreak strain of O104:H4 (BAA-2326) and Montana outbreak strain of O104:H21 (BAA-178). All O104 strains, cattle or human, were negative for *eae* (intimin) and for genes associated with type III secretory system (*esc*R, *esp*A, *esp*D, *esc*D, *esc*R, *esp*D, *esp*B, *ler*, *esc*T, *sep*L, *sep*Q, and *tir*).

### Cattle O104:H7

**Fig 1:**
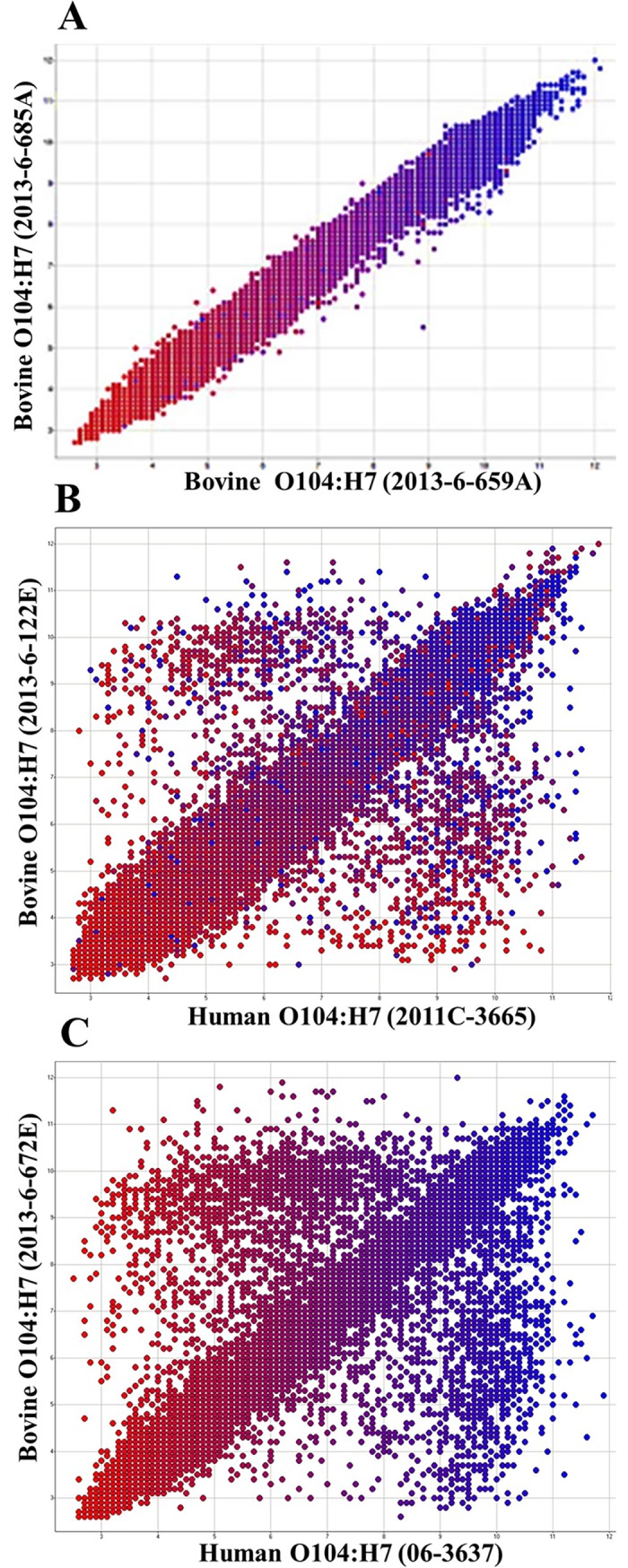
Scatter plots of pairwise comparisons between *Escherichia coli* O104:H7 strains of bovine and human origin. Each dot represents the robust multiarray average (RMA)-summarized probe set intensity. Dots clustered on the diagonal line represent genes present in both genomes; scattered dots indicate genome-specific probe sets: upper-left for the genes represented by the Y-axis, and lower-right for the genes represented by the X-axis. Dots in the plots are colored red to blue (2.6 to 12) based on the normalized RMA intensity values using human clinical O104:H7 (06–3637) as the reference strain.

All bovine O104:H7 strains were positive for the following virulence genes characteristic of STEC pathotype: *stx*1 (subtype c), *terD* (tellurite resistance protein), and *iha* (IrgA homolog adhesin). Enterohemolysin (*ehxA*) was present in four of the six bovine strains. The strains were also positive for adhesins, *lpfA* (long polar fimbriae) and *fimH* (mannose specific adhesin); and antimicrobial resistance genes, such as *ampH* (penicillin binding protein), *marC* (multiple antibiotic resistance protein), and *pmrD* (polymyxin resistance protein) (Table 1). The six cattle O104 strains were closely related (r = 0.86–0.98; Table 2) to each other, except for a few differences in phage related and non-annotated genes (data not shown). Scatter plot data of the strain pairs revealed that three bovine O104:H7 strains, 2013-6-659A, 2013-6-672E and 2013-6-685A, isolated from the same feedlot, were almost indistinguishable (Fig 1A).

**Table 1:** Comparison of major virulence genes of bovine *E*. *coli* O104:H7 with human O104:H7 strains based on microarray.

		Bovine O104:H7 strains						Human O104:H7 strains				
Genes	Product	2013-6-659A	2013-6-672E	2013-6-685A	2013-6-48C	2013-6-122E	2013-6-148B	06–3637	07–3598	08–4061	2011C-3665	2012C-3400
**Enterohemorrhagic *E*. *coli* (EHEC)**												
*stx*1	Shiga toxin 1	+	+	+	+	+	+				+	
*stx*2	Shiga toxin 2							+		+		+
*terD*	Tellurite resistance protein	+	+	+	+	+	+					
*ehxA*	Enterohemolysin	+	+			+	+	+	+	+		+
*sub*	Subtilase cytotoxin							+	+	+		+
*iha*	IrgA homologue adhesion	+	+	+	+	+	+					
*saa*	Shiga toxin-producing *E*. *coli* autoagglutinating adhesin							+	+	+		+
**Other genes**												
*ampH*	Penicillin binding protein	+	+	+	+	+	+	+	+	+	+	+
*lpfA*	Long polar fimbriae	+	+	+	+	+	+	+	+	+	+	+
*fimH*	Mannose specific adhesin	+	+	+	+	+	+	+	+	+	+	+
*pmrD*	Polymyxin resistance protein	+	+	+	+	+	+	+	+	+	+	+
*marC*	Multiple antibiotic resistance protein	+	+	+	+	+	+	+	+	+	+	+
*espP*	Extracellular serine protease							+	+	+		+

**Table 2:** Pearson correlation analysis of cattle O104:H7 and human O104:H7 strains.

	Cattle O104:H7						Human O104:H7				
Strains	2013-6-659A	2013-6-672E	2013-6-685A	2013-6-48C	2013-6-122E	2013-6-148B	06–3637	07–3598	08–4061	2011C-3665	2012C-3400
**Bovine O104:H7**											
**2013-6-659A**	1	0.98	0.94	0.94	0.91	0.9	0.78	0.78	0.77	0.83	0.76
**2013-6-672E**	0.98	1	0.95	0.94	0.92	0.9	0.77	0.78	0.77	0.83	0.76
**2013-6-685A**	0.94	0.95	1	0.91	0.91	0.91	0.77	0.77	0.77	0.85	0.76
**2013-6-48C**	0.94	0.94	0.91	1	0.89	0.86	0.77	0.77	0.76	0.81	0.75
**2013-6-122E**	0.91	0.92	0.91	0.89	1	0.93	0.78	0.79	0.78	0.85	0.78
**2013-6-148B**	0.90	0.90	0.91	0.86	0.93	1	0.77	0.78	0.77	0.84	0.77
**Human O104:H7**											
**06–3637**	0.78	0.77	0.77	0.77	0.78	0.77	1	0.94	0.93	0.78	0.91
**07–3598**	0.78	0.78	0.77	0.77	0.79	0.78	0.94	1	0.95	0.78	0.93
**08–4061**	0.77	0.77	0.77	0.76	0.78	0.77	0.93	0.95	1	0.78	0.93
**2011C-3665**	0.83	0.83	0.85	0.81	0.85	0.84	0.78	0.78	0.78	1	0.77
**2012C-3400**	0.76	0.76	0.76	0.75	0.78	0.77	0.91	0.93	0.93	0.77	1

### Cattle O104:H7 vs. Human O104:H7

**Fig 2:**
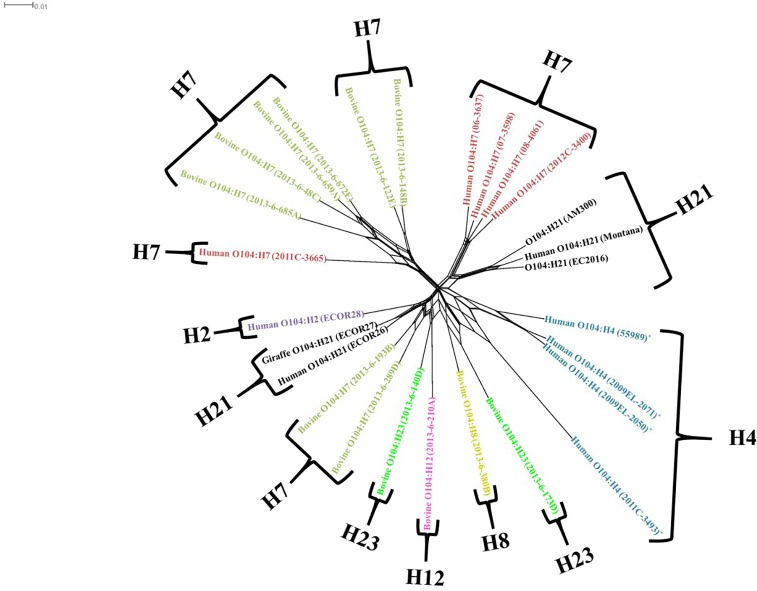
Splits tree representation of O104 diversity in our collections. Phylogenetic analysis of 33 O104 strains using the FDA-ECID gene differences data from all the probe sets. The tree was developed using the Neighbor net algorithm using Neighbor joining method (SplitsTree4). Microarray analysis of these strains was able to reveal several large parallel paths, indicative of phylogenetic incompatibilities due to probable recombination. The scale bar represents a 0.01 base substitution (bp) per site.

Of the five human O104:H7 strains, three were positive for *stx*2, one was positive for *stx*1 (subtype c) and another was negative for both. Virulence genes such as *ehxA*, *ampH*, *lpfA*, *fimH*, *pmrD*, and *marC* were present in both human and bovine O104:H7 strains (Table 1). The human strains contained the following virulence genes that were absent in the bovine strains: *sub* (4/5 strains; encodes for subtilase cytotoxin), *esp*P (4/5 strains; encodes for extracellular serine protease), and *saa* (4/5 strains; encodes for STEC auto agglutinating adhesin). Some of the virulence genes present in bovine O104:H7 strains, such as *ter*D and *iha* were absent in human O104:H7 strains. One human clinical (O104:H7; 2011C-3665) strain was more closely related to bovine O104:H7 strains (r = 0.81–0.85; Table 2; Fig 1B; Fig 2) than the other four human strains (Fig 1C; Fig 2; S1 Fig). The human strain was positive for *stx*1c, *lpfA*, *ampH*, *fimH*, *marC*, *pmrD* and negative for other virulence genes such as *stx*2, *sub*, *saa*, and *espP*, similar to the bovine O104:H7 strains, however, it was negative for *terD*, *ehxA* and *iha* unlike the bovine O104:H7 strains. The human strain, 2011C-3665, had the lowest number of probe sets (3,344 to 4,024) different from any of the bovine strains. Comparison of bovine O104:H7 strains with the other four human clinical (O104:H7) strains revealed probe set differences ranging from 3,481 to 5,412.

### Cattle O104:H7 vs. human O104:H4 and O104:H21

Virulence genes characteristic of enteroaggregative *E*. *coli* (*pet*- Per-activated serine protease autotransporter enterotoxin, *aatA*- EAEC virulence plasmid (pAA), *aggR*- Transcriptional regulator, *pic*-Protein involved in intestinal colonization, and *aatP*- permease) were present in the German outbreak (O104:H4) strain (S1 Table), but absent in O104:H7 and O104:H21 strains. Extracellular serine protease (*espP*) was present only in the Montana outbreak strain, but absent in German outbreak and bovine O104:H7 strains. The bovine O104:H7 strains and the German O104:H4 strain were positive for *terD*. Enterohemolysin gene (*ehxA*), absent in the O104:H4 strain, was present in four bovine O104:H7 strains and in the O104:H21 strain. Some of the virulence genes (*ampH*, *pmrD*, *marC*, and *lpfA*) detected in bovine O104:H7 strains and human clinical O104:H7 strains were also present in O104:H4 and O104:H21 strains (Table 1; S1 Table). The complete list of genes detected in bovine *E*. *coli* O104:H7 and human O104:H4 and O104:H21 outbreak strains is shown in S1 Table.

### Phylogenetic relationship

**Fig 3:**
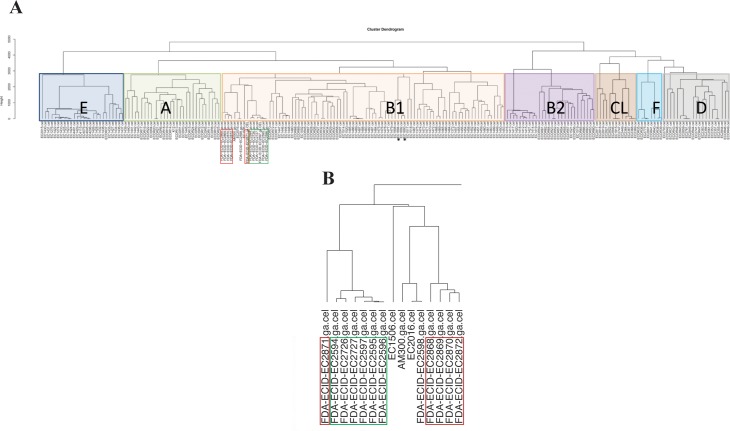
Genotype analysis of the O104 strains from bovine and clinical origins. (A) Hierarchical cluster dendrogram generated by array probeset differences for 241 strains including the diarrheagenic *Escherichia coli* collection representing the broad diversity across the *E*. *coli* species. Phylogroups are highlighted with color shading. The strains reported in this study from human clinical and bovine origin are highlighted in red and green boxes, respectively. Additionally, O104:H4 strains from the German and Georgian outbreaks [15] (indicated with asterisk) are used as reference. The scale bar represents the number of probeset differences. (B) Enlargement of the cluster region containing bovine and human *E*. *coli* O104:H7 strains from our study.

To gain perspective on the importance of O104 with regard to pathogenicity potential, we analyzed its relationship to the observed diversity in the *E*. *coli* species using the pan genome array. *E*. *coli* segregate into six observable phylogroups (A, B1, B2, CL, D, E, and F) as reported previously [14]. All strains analyzed in this study belonged to the B1 phylogroup (Fig 3A). However, O104:H4 strains of the German and Georgian outbreaks of 2011 and 2009 [15], respectively (Fig 3A noted with asterisks), are distant from either the clinical or bovine O104:H7 strains (denoted in red and green boxes, respectively). The most closely related strain at a pan genome level, to four out of five clinical strains in this study was the O104:H21 strain isolated from bovine milk samples in Montana. The other clinical strain clustered with the bovine strains and this collective group was most closely related to a *stx*1 and 2 positive O146:H21 diarrheagenic *E*. *coli* strain.

As a corollary, we compared all O104 strains in our collection belonging to different serotypes independently to measure genomic networking and presumed recombination (Fig 2) which separated into 8 general groups. Similar to the hierarchical clustering method, one clinical O104:H7 strain (2011C-3665) clustered with the bovine O104:H7 STEC strains showing some parallel recombinatory paths. Likewise, O104 Georgian and German strains were distinctly separate groups. Regardless, given the instances of virulence markers noted above (Table 1) and observed relationships to known pathogens, O104:H7 is an important serotype to monitor for significant human pathogenicity potential.

## Discussion

Several molecular methods are available for the genetic characterization of foodborne pathogens, including Shiga toxin-producing *E*. *coli*. Molecular techniques, such as DNA microarray and whole genome sequencing (WGS), allow genome-wide characterization of the organisms. The limitation of the microarray is that it allows the detection of only specific gene targets, unlike WGS, which enables the detection of novel genes in the target organisms. However, WGS is time-consuming and laborious compared to microarray [14, 16]. Custom DNA microarray detecting multitude of genes, designed using published whole genome sequences of *E*. *coli* and *Shigella*, enable rapid genome-scale analysis of pathogenic *E*. *coli* [14, 17–19].

All the bovine O104:H7 strains investigated in this study were negative for *stx*2, which is more frequently associated with human illnesses, particularly hemolytic uremic syndrome, than *stx*1 [20]. The bovine O104:H7 strains and one of the five human O104:H7 strain were positive for *stx*1 of subtype 1c. Other STEC serogroups carrying *stx*1c have been associated with diarrhea and in a few cases serious complications in human patients [21]. For example, STEC O78:H- carrying *stx*1c was reported to be the causative agent of HUS in a 2-week old boy in Finland [22]. Only human clinical O104:H7 (3/5) and both human outbreak strains (O104:H4 and O104:H21) carried *stx*2. However, *stx*2 subtype was not identified in our study, because the array cannot differentiate the *stx*2 subtypes [14]. All O104:H7 strains, both bovine and human, similar to O104:H4 and O104:H21 outbreak strains [9, 11, 23], were negative for intimin (*eae*), an adhesin involved in the attachment to the host cells [24]. However, O104:H7 carried other adhesins, such as IrgA homologue adhesion (*iha*), mannose-specific adhesin (*fimH*), and long polar fimbriae (*lpfA*), which are involved in adhesion of bacteria to the host epithelial cells [25–27]. These adhesins may be involved in the attachment of the bacteria to the host cells in the absence of intimin. Long polar fimbriae (*lpfA*), which is also present in German outbreak strain (O104:H4), has been reported to be involved in initial attachment and colonization of the intestine [26]. Enterohemolysin (*ehxA*) was present in four of the bovine strains and in all human O104:H7 strains, except one, and in Montana outbreak strain (O104:H21). Previous studies have reported the association of *ehxA* with diarrhea and hemolytic uremic syndrome [28]. Cheng et al. (2015) have reported that hemolysin encoded by *ehxA* in *E*. *coli* O157:H7 is involved in cytotoxicity of macrophages and release of IL-1β in humans [29]. All bovine O104:H7 strains and the German outbreak strain were positive for *terD*, which codes for tellurite resistance protein. Shiga toxin-producing *E*. *coli* serogroups such as O26, O45, O111 and O157 carry tellurite resistance gene [30]. Tellurite resistance gene has been reported to be involved in the survival of bacteria by conferring protection against host defense [31].

Although there was some similarity in the virulence gene profile of human and bovine O104 strains, the key virulence genes, such as *stx*2 responsible for more serious infections, including hemolytic uremic syndrome, was absent in bovine strains, but present in human clinical and outbreak strains. *Escherichia coli* O104:H4 responsible for food-borne outbreak in Germany has been reported to be evolved by the uptake of *stx*2 encoding bacteriophages [32]. Similarly, bovine O104:H7 strains could evolve into highly pathogenic strains by acquiring other virulence genes responsible for human illnesses by gain and loss of genomic islands, prophages, and plasmids. All the strains investigated in the present study belong to B1 phylogroup. Most *E*. *coli* strains isolated from beef cattle carrying virulence genes (*hlyA*, *stx*1, *stx*2, *eae*) belonged to this phylogroup [33]. Phylogenetic analysis of STEC strains has revealed that majority (70%) of them belonged to B1 phylogroup [34].

Based on the scatterplots, splits tree, cluster dendrogram and Pearson correlation coefficients; all the bovine O104:H7 strains were closely related to each other, and one of the five human clinical strains (O104:H7; 2011C-3665) included in the study was also more closely related with the bovine strains. The close genetic relatedness of the human clinical strain (O104:H7; 2011C-3665) and bovine O104:H7 strains is further explained by fewer probe set differences (3,344 to 4,024) between them, they also showed similarity in their virulence gene profile. These findings indicate that the human clinical strain (2011C-3665) and the bovine strains most likely shared similar environment. This suggests that cattle feces could potentially be a source of O104:H7 strains associated with diarrhea in humans. Additionally, H-type appears to be more important than O-type as an attribute in relating all of the clinical and bovine strains included in this study based on the phylogenetic analysis. Similar finding was reported on O104 STEC strains based on pulsed-field gel electrophoresis [13]. Ju et al. (2012) hypothesized that *E*. *coli* serogroups carrying same flagellar antigens share common ancestors based on phylogenetic analysis of whole genome sequences of non-O157 *E*. *coli* serogroups [35]. We compared bovine and human O104:H7 strains to human outbreak strains of O104 belonging to different serotypes (O104:H4 and O104:H21) to determine similarities. However, our study revealed that the outbreak strains are distantly related to bovine and human O104:H7 strains, except for a few virulence genes common among all the strains.

In conclusion, microarray-based analyses of genes of bovine O104:H7 strains isolated from cattle feces suggest the potential of the serotype to be a diarrheagenic human pathogen. Because cattle harbor these strains and shed them in their feces, O104:H7 strains also have the potential to be foodborne pathogens to humans.

## Supporting information

S1 Table Comparison of major virulence genes of bovine *E*. *coli* O104:H7 strains with human O104:H4 and O104:H21 outbreak strains based on microarray. (DOCX)

S2 Table Pearson correlation analysis of the cattle *E*. *coli* O104:H7 and human O104:H4 and O104:H21 strains. (DOCX)

S1 Fig Scatter plots pairwise comparisons between *Escherichia coli* O104:H7 strains of bovine and human origin. (A) Comparison of bovine O104:H7 strain (2013-6-48C) with human O104:H7 strains (B) Comparison of bovine O104:H7 strain (2013-6-122E) with human O104:H7 strains (C) Comparison of bovine O104:H7 strain (2013-6-148B) with human O104:H7 strains (D) Comparison of bovine O104:H7 strain (2013-6-659A) with human O104:H7 strains (E) Comparison of bovine O104:H7 strain (2013-6-672E) with human O104:H7 strains (F) Comparison of bovine O104:H7 strain (2013-6-685A) with human O104:H7 strains
(TIF)

S1 Excel File Robust MultiArray Averaging (RMA) summarized probe set intensity data of bovine and human *E*. *coli* O104:H7 strains. (XLSX)

## References

[1] Ahmed SA, Awosika J, Baldwin C, Bishop-Lilly KA, Biswas B, Broomall S, et al Genomic Comparison of *Escherichia coli* O104:H4 Isolates from 2009 and 2011 Reveals Plasmid, and Prophage Heterogeneity, Including Shiga Toxin Encoding Phage *stx*2. PloS one. 2012;7(11):e48228 doi: 10.1371/journal.pone.0048228 PubMed PMID: PMC3486847. 23133618 10.1371/journal.pone.0048228 PMC3486847

[2] Brzuszkiewicz E, Thurmer A, Schuldes J, Leimbach A, Liesegang H, Meyer FD, et al Genome sequence analyses of two isolates from the recent *Escherichia coli* outbreak in Germany reveal the emergence of a new pathotype: Entero-Aggregative-Haemorrhagic *Escherichia coli* (EAHEC). Archives of microbiology. 2011;193(12):883–91. doi: 10.1007/s00203-011-0725-6 ; PubMed Central PMCID: PMC3219860. 21713444 10.1007/s00203-011-0725-6 PMC3219860

[3] Mellmann A, Harmsen D, Cummings CA, Zentz EB, Leopold SR, Rico A, et al Prospective Genomic Characterization of the German Enterohemorrhagic *Escherichia coli* O104:H4 Outbreak by Rapid Next Generation Sequencing Technology. PloS one. 2011;6(7):e22751 doi: 10.1371/journal.pone.0022751 21799941 10.1371/journal.pone.0022751 PMC3140518

[4] Rasko DA, Webster DR, Sahl JW, Bashir A, Boisen N, Scheutz F, et al Origins of the *E*. *coli* strain causing an outbreak of hemolytic-uremic syndrome in Germany. N Engl J Med. 2011;365 doi: 10.1056/NEJMoa1106920 21793740 10.1056/NEJMoa1106920 PMC3168948

[5] Rohde H, Qin J, Cui Y, Li D, Loman NJ, Hentschke M, et al Open-Source Genomic Analysis of Shiga-Toxin–Producing *E*. *coli* O104:H4. New England Journal of Medicine. 2011;365(8):718–24. doi: 10.1056/NEJMoa1107643 21793736 10.1056/NEJMoa1107643

[6] Paddock ZD, Bai J, Shi X, Renter DG, Nagaraja TG. Detection of *Escherichia coli* O104 in the feces of feedlot cattle by a multiplex PCR assay designed to target major genetic traits of the virulent hybrid strain responsible for the 2011 German outbreak. Applied and environmental microbiology. 2013;79(11):3522–5. doi: 10.1128/AEM.00246-13 ; PubMed Central PMCID: PMCPMC3648041. 23542615 10.1128/AEM.00246-13 PMC3648041

[7] Wieler LH, Semmler T, Eichhorn I, Antao EM, Kinnemann B, Geue L, et al No evidence of the Shiga toxin-producing *E*. *coli* O104:H4 outbreak strain or enteroaggregative *E*. *coli* (EAEC) found in cattle faeces in northern Germany, the hotspot of the 2011 HUS outbreak area. Gut pathogens. 2011;3(1):17 doi: 10.1186/1757-4749-3-17 ; PubMed Central PMCID: PMC3227623. 22051440 10.1186/1757-4749-3-17 PMC3227623

[8] Auvray F, Dilasser F, Bibbal D, Kerouredan M, Oswald E, Brugere H. French cattle is not a reservoir of the highly virulent enteroaggregative Shiga toxin-producing *Escherichia coli* of serotype O104:H4. Veterinary microbiology. 2012;158(3–4):443–5. doi: 10.1016/j.vetmic.2012.02.029 . 22424867 10.1016/j.vetmic.2012.02.029

[9] Shridhar PB, Noll LW, Shi X, Cernicchiaro N, Renter DG, Bai J, et al *Escherichia coli* O104 in Feedlot Cattle Feces: Prevalence, Isolation and Characterization. PloS one. 2016;11(3):e0152101 doi: 10.1371/journal.pone.0152101 27010226 10.1371/journal.pone.0152101 PMC4807062

[10] Rump LV, Bodeis-Jones S, Abbott J, Zhao S, Kase J, Lorenz S, et al Genetic characterization of *Escherichia coli* O104 isolates from different sources in the United States. Applied and environmental microbiology. 2012;78(5):1615–8. doi: 10.1128/AEM.07533-11 ; PubMed Central PMCID: PMC3294489. 22210209 10.1128/AEM.07533-11 PMC3294489

[11] Feng P, Weagant SD, Monday SR. Genetic analysis for virulence factors in *Escherichia coli* O104:H21 that was implicated in an outbreak of hemorrhagic colitis. Journal of clinical microbiology. 2001;39(1):24–8. doi: 10.1128/JCM.39.1.24-28.2001 ; PubMed Central PMCID: PMCPMC87673. 11136742 10.1128/JCM.39.1.24-28.2001 PMC87673

[12] Delannoy S, Beutin L, Burgos Y, Fach P. Specific detection of enteroaggregative hemorrhagic *Escherichia coli* O104:H4 strains by use of the CRISPR locus as a target for a diagnostic real-time PCR. Journal of clinical microbiology. 2012;50(11):3485–92. doi: 10.1128/JCM.01656-12 ; PubMed Central PMCID: PMC22895033. 22895033 10.1128/JCM.01656-12 PMC3486251

[13] Miko A, Delannoy S, Fach P, Strockbine NA, Lindstedt BA, Mariani-Kurkdjian P, et al Genotypes and virulence characteristics of Shiga toxin-producing *Escherichia coli* O104 strains from different origins and sources. International journal of medical microbiology: IJMM. 2013;303(8):410–21. doi: 10.1016/j.ijmm.2013.05.006 . 23777812 10.1016/j.ijmm.2013.05.006

[14] Patel IR, Gangiredla J, Lacher DW, Mammel MK, Jackson SA, Lampel KA, et al FDA-*Escherichia coli* Identification (FDA-ECID) Microarray: A Pan-Genome Molecular Toolbox for Serotyping, Virulence Profiling, Molecular Epidemiology, and Phylogeny. Applied and environmental microbiology. 2016;82(11):3384–94. doi: 10.1128/AEM.04077-15 27037122 10.1128/AEM.04077-15 PMC4959244

[15] Jackson SA, Kotewicz ML, Patel IR, Lacher DW, Gangiredla J, Elkins CA. Rapid Genomic-Scale Analysis of *Escherichia coli* O104:H4 by Using High-Resolution Alternative Methods to Next-Generation Sequencing. Applied and environmental microbiology. 2012;78(5):1601–5. doi: 10.1128/AEM.07464-11 22210216 10.1128/AEM.07464-11 PMC3294476

[16] Joensen KG, Scheutz F, Lund O, Hasman H, Kaas RS, Nielsen EM, et al Real-Time Whole-Genome Sequencing for Routine Typing, Surveillance, and Outbreak Detection of Verotoxigenic Escherichia coli. Journal of clinical microbiology. 2014;52(5):1501–10. doi: 10.1128/JCM.03617-13 24574290 10.1128/JCM.03617-13 PMC3993690

[17] Baranzoni GM, Fratamico PM, Gangiredla J, Patel I, Bagi LK, Delannoy S, et al Characterization of Shiga Toxin Subtypes and Virulence Genes in Porcine Shiga Toxin-Producing *Escherichia coli*. Front Microbiol. 2016;7:574 Epub 2016/05/06. doi: 10.3389/fmicb.2016.00574 ; PubMed Central PMCID: PMCPMC4838603. 27148249 10.3389/fmicb.2016.00574 PMC4838603

[18] Lacher DW, Gangiredla J, Jackson SA, Elkins CA, Feng PCH. Novel Microarray Design for Molecular Serotyping of Shiga Toxin-Producing *Escherichia coli* Strains Isolated from Fresh Produce. Applied and environmental microbiology. 2014;80(15):4677–82. doi: 10.1128/AEM.01049-14 24837388 10.1128/AEM.01049-14 PMC4148803

[19] Lacher DW, Gangiredla J, Patel I, Elkins CA, Feng PCH. Use of the *Escherichia coli* Identification Microarray for Characterizing the Health Risks of Shiga Toxin–Producing *Escherichia coli* Isolated from Foods. Journal of food protection. 2016;79(10):1656–62. doi: 10.4315/0362-028X.JFP-16-176 28221838 10.4315/0362-028X.JFP-16-176

[20] Siegler RL, Obrig TG, Pysher TJ, Tesh VL, Denkers ND, Taylor FB. Response to Shiga toxin 1 and 2 in a baboon model of hemolytic uremic syndrome. Pediatric nephrology. 2003;18(2):92–6. doi: 10.1007/s00467-002-1035-7 . 12579394 10.1007/s00467-002-1035-7

[21] Zhang W, Bielaszewska M, Kuczius T, Karch H. Identification, Characterization, and Distribution of a Shiga Toxin 1 Gene Variant (stx1c) in Escherichia coli Strains Isolated from Humans. Journal of clinical microbiology. 2002;40(4):1441–6. doi: 10.1128/JCM.40.4.1441-1446.2002 11923370 10.1128/JCM.40.4.1441-1446.2002 PMC140390

[22] Lienemann T, Salo E, Rimhanen-Finne R, Ronnholm K, Taimisto M, Hirvonen JJ, et al Shiga toxin-producing *Escherichia coli* serotype O78:H(-) in family, Finland, 2009. Emerging infectious diseases. 2012;18(4):577–81. Epub 2012/04/04. doi: 10.3201/eid1804.111310 ; PubMed Central PMCID: PMCPMC3309701. 22469631 10.3201/eid1804.111310 PMC3309701

[23] Bielaszewska M, Mellmann A, Zhang W, Köck R, Fruth A, Bauwens A, et al Characterisation of the *Escherichia coli* strain associated with an outbreak of haemolytic uraemic syndrome in Germany, 2011: a microbiological study. The Lancet Infectious Diseases. 2011;11(9):671–6. doi: 10.1016/S1473-3099(11)70165-7 21703928 10.1016/S1473-3099(11)70165-7

[24] Jerse AE, Yu J, Tall BD, Kaper JB. A genetic locus of enteropathogenic *Escherichia coli* necessary for the production of attaching and effacing lesions on tissue culture cells. Proceedings of the National Academy of Sciences of the United States of America. 1990;87(20):7839–43. ; PubMed Central PMCID: PMCPMC54845. 2172966 10.1073/pnas.87.20.7839 PMC54845

[25] Tarr PI, Bilge SS, Vary JC, Jelacic S, Habeeb RL, Ward TR, et al Iha: a novel *Escherichia coli* O157:H7 adherence-conferring molecule encoded on a recently acquired chromosomal island of conserved structure. Infection and immunity. 2000;68(3):1400–7. ; PubMed Central PMCID: PMCPMC97294. 10678953 10.1128/iai.68.3.1400-1407.2000 PMC97294

[26] Ross BN, Rojas-Lopez M, Cieza RJ, McWilliams BD, Torres AG. The Role of Long Polar Fimbriae in *Escherichia coli* O104:H4 Adhesion and Colonization. PloS one. 2015;10(10):e0141845 doi: 10.1371/journal.pone.0141845 26517878 10.1371/journal.pone.0141845 PMC4636846

[27] Wold AE, Thorssen M, Hull S, Eden CS. Attachment of *Escherichia coli* via mannose- or Gal alpha 1—-4Gal beta-containing receptors to human colonic epithelial cells. Infection and immunity. 1988;56(10):2531–7. Epub 1988/10/01. ; PubMed Central PMCID: PMCPMC259607. 2901402 10.1128/iai.56.10.2531-2537.1988 PMC259607

[28] Schmidt H, Karch H. Enterohemolytic phenotypes and genotypes of shiga toxin-producing *Escherichia coli* O111 strains from patients with diarrhea and hemolytic-uremic syndrome. Journal of clinical microbiology. 1996;34(10):2364–7. ; PubMed Central PMCID: PMC8880480. 8880480 10.1128/jcm.34.10.2364-2367.1996 PMC229269

[29] Cheng Y-L, Song L-Q, Huang Y-M, Xiong Y-W, Zhang X-A, Sun H, et al Effect of enterohaemorrhagic *Escherichia coli* O157:H7-specific enterohaemolysin on interleukin-1β production differs between human and mouse macrophages due to the different sensitivity of NLRP3 activation. Immunology. 2015;145(2):258–67. doi: 10.1111/imm.12442 ; PubMed Central PMCID: PMCPMC4427390. 25580516 10.1111/imm.12442 PMC4427390

[30] Orth D, Grif K, Dierich MP, Wurzner R. Variability in tellurite resistance and the ter gene cluster among Shiga toxin-producing *Escherichia coli* isolated from humans, animals and food. Research in microbiology. 2007;158(2):105–11. doi: 10.1016/j.resmic.2006.10.007 . 17317110 10.1016/j.resmic.2006.10.007

[31] Valková D, Valkovičová L, Vávrová S, Kováčová E, Mravec J, Turňa J. The contribution of tellurite resistance genes to the fitness of *Escherichia coli* uropathogenic strains. centeurjbiol. 2007;2(2):182–91. doi: 10.2478/s11535-007-0019-9

[32] Beutin L, Hammerl JA, Reetz J, Strauch E. Shiga toxin-producing *Escherichia coli* strains from cattle as a source of the Stx2a bacteriophages present in enteroaggregative *Escherichia coli* O104:H4 strains. International journal of medical microbiology: IJMM. 2013;303(8):595–602. doi: 10.1016/j.ijmm.2013.08.001 . 24012149 10.1016/j.ijmm.2013.08.001

[33] Unno T, Han D, Jang J, Lee S-N, Ko G, Choi HY, et al Absence of *Escherichia coli* Phylogenetic Group B2 Strains in Humans and Domesticated Animals from Jeonnam Province, Republic of Korea. Applied and environmental microbiology. 2009;75(17):5659–66. doi: 10.1128/AEM.00443-09 PubMed PMID: PMC2737926. 19592524 10.1128/AEM.00443-09 PMC2737926

[34] Girardeau JP, Dalmasso A, Bertin Y, Ducrot C, Bord S, Livrelli V, et al Association of Virulence Genotype with Phylogenetic Background in Comparison to Different Seropathotypes of Shiga Toxin-Producing Escherichia coli Isolates. Journal of clinical microbiology. 2005;43(12):6098–107. doi: 10.1128/JCM.43.12.6098-6107.2005 PubMed PMID: PMC1317181. 16333104 10.1128/JCM.43.12.6098-6107.2005 PMC1317181

[35] Ju W, Cao G, Rump L, Strain E, Luo Y, Timme R, et al Phylogenetic Analysis of Non-O157 Shiga Toxin-Producing *Escherichia coli* Strains by Whole-Genome Sequencing. Journal of clinical microbiology. 2012;50(12):4123–7. doi: 10.1128/JCM.02262-12 23052305 10.1128/JCM.02262-12 PMC3502965

