# Supplementary material for: DNA microarray-based assessment of virulence potential of Shiga toxin gene-carrying Escherichia coli O104:H7 isolated from feedlot cattle feces
Source: PLoS One. 2018 Apr 30;13(4):e0196490. doi: 10.1371/journal.pone.0196490 (PMC5927410; doi:10.1371/journal.pone.0196490)
Supplement: S1 Table — (DOCX) [file pone.0196490.s001.docx]

**S1 Table: Comparison of major virulence genes of bovine *E. coli* O104:H7 strains with human O104:H4 and O104:H21 outbreak strains based on microarray**

|  |  | **Human strains** | |  | **Bovine O104:H7 strains** | | | | | |  |
| --- | --- | --- | --- | --- | --- | --- | --- | --- | --- | --- | --- |
| **Genes** | **Product** | **O104:H4 (German)** | **O104:H21 (Montana)** |  | **2013-6-659A** | **2013-6-672E** | **2013-6-685A** | **2013-6-48C** | **2013-6-122E** | **2013-6-148B** | |
| **Enterohemorrhagic *E. coli* (EHEC)** | | | | | | | | | | |  |
| *stx*1 | Shiga toxin 1 |  |  |  | + | + | + | + | + | + |  |
| *stx*2 | Shiga toxin 2 | + | + |  |  |  |  |  |  |  |  |
| *terD* | Tellurite resistance protein | + |  |  | + | + | + | + | + | + |  |
| *ehxA* | Enterohemolysin |  | + |  | + | + |  |  | + | + |  |
| *sub* | Subtilase cytotoxin |  | + |  |  |  |  |  |  |  |  |
| *iha* | IrgA homologue adhesin | + | + |  | + | + | + | + | + | + |  |
| *saa* | Shiga toxin producing *E. coli* autoagglutinating adhesin |  | + |  |  |  |  |  |  |  |  |
| **Enteroaggregative *E. coli* (EAEC)** | | | | | | | | | | |  |
| *pet* | Per-activated serine protease autotransporter enterotoxin EspC | + |  |  |  |  |  |  |  |  |  |
| *aatA* | EAEC virulence plasmid (pAA) | + |  |  |  |  |  |  |  |  |  |
| *aggR* | Transcriptional regulator | + |  |  |  |  |  |  |  |  |  |
| *pic* | Protein involved in intestinal colonisation | + |  |  |  |  |  |  |  |  |  |
| *aatP* | AatP permease | + |  |  |  |  |  |  |  |  |  |
| **Other genes** | | | | | | | | | | |  |
| *ampH* | Penicillin binding protein | + | + |  | + | + | + | + | + | + |  |
| *lpfA* | Long polar fimbriae | + | + |  | + | + | + | + | + | + |  |
| *pmrD* | Polymyxin resistance protein | + | + |  | + | + | + | + | + | + |  |
| *marC* | Multiple antibiotic resistance protein | + | + |  | + | + | + | + | + | + |  |
| *fimH* | FimH protein precursor (mannose-specific adhesin) |  | + |  | + | + | + | + | + | + |  |
| *esp*P | Extracellular serine protease |  | **+** |  |  |  |  |  |  |  |  |
| *mchC* | MchC Protein | + |  |  |  |  |  |  |  |  |  |
| *fyuA* | Iron acquisition outer membrane yersiniabactin receptor | + |  |  |  |  |  |  |  |  |  |
