# Supplementary material for: DNA microarray-based assessment of virulence potential of Shiga toxin gene-carrying Escherichia coli O104:H7 isolated from feedlot cattle feces
Source: PLoS One. 2018 Apr 30;13(4):e0196490. doi: 10.1371/journal.pone.0196490 (PMC5927410; doi:10.1371/journal.pone.0196490)
Supplement: S2 Table — (DOCX) [file pone.0196490.s002.docx]

**S2 Table. Pearson correlation analysis of the cattle *E. coli* O104:H7 and human O104:H4 and O104:H21 strains**

|  | **Pearson correlation coefficients** | | | | | | | | |
| --- | --- | --- | --- | --- | --- | --- | --- | --- | --- |
| **Strains** | **Human strains** | |  | **Cattle O104:H7 strains** | | | | | |
|  | **O104:H4 (BAA-2326)** | **O104:H21 (BAA-178)** |  | **2013-6-659A** | **2013-6-672E** | **2013-6-685A** | **2013-6-48C** | **2013-6-122E** | **2013-6-148B** |
| **Human O104:H4 (BAA-2326 )** | 1 | 0.74 |  | 0.72 | 0.72 | 0.73 | 0.72 | 0.75 | 0.75 |
| **Human O104:H21 (BAA-178)** | 0.74 | 1 |  | 0.79 | 0.79 | 0.78 | 0.78 | 0.77 | 0.76 |
| **Cattle O104:H7 (2013-6-659A)** | 0.72 | 0.79 |  | 1 | 0.98 | 0.95 | 0.94 | 0.91 | 0.90 |
| **Cattle O104:H7 (2013-6-672E)** | 0.72 | 0.79 |  | 0.98 | 1 | 0.95 | 0.94 | 0.92 | 0.90 |
| **Cattle O104:H7 (2013-6-685A)** | 0.73 | 0.78 |  | 0.95 | 0.95 | 1 | 0.91 | 0.91 | 0.91 |
| **Cattle O104:H7 (2013-6-48C)** | 0.72 | 0.78 |  | 0.94 | 0.94 | 0.91 | 1 | 0.89 | 0.86 |
| **Cattle O104:H7 (2013-6-122E)** | 0.75 | 0.77 |  | 0.91 | 0.92 | 0.91 | 0.89 | 1 | 0.93 |
| **Cattle O104:H7 (2013-6- 148B)** | 0.75 | 0.76 |  | 0.90 | 0.90 | 0.91 | 0.86 | 0.93 | 1 |
