# Supplementary figures and images for: DNA microarray-based assessment of virulence potential of Shiga toxin gene-carrying Escherichia coli O104:H7 isolated from feedlot cattle feces
Source: PLoS One. 2018 Apr 30;13(4):e0196490. doi: 10.1371/journal.pone.0196490 (PMC5927410; doi:10.1371/journal.pone.0196490)

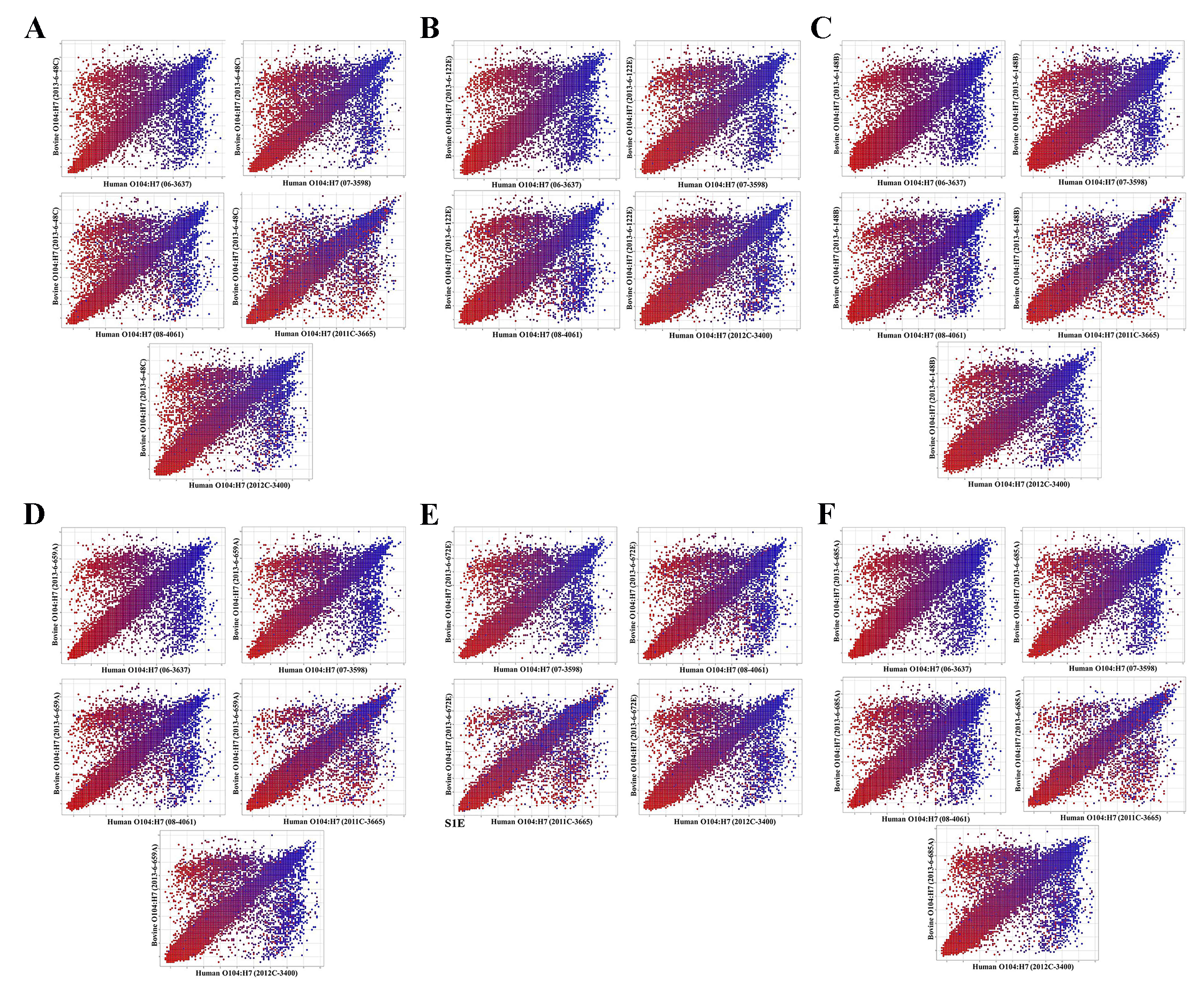

Supplement: S1 Fig — (A) Comparison of bovine O104:H7 strain (2013-6-48C) with human O104:H7 strains (B) Comparison of bovine O104:H7 strain (2013-6-122E) with human O104:H7 strains (C) Comparison of bovine O104:H7 strain (2013-6-148B) with human O104:H7 strains (D) Comparison of bovine O104:H7 strain (2013-6-659A) with human O104:H7 strains (E) Comparison of bovine O104:H7 strain (2013-6-672E) with human O104:H7 strains (F) Comparison of bovine O104:H7 strain (2013-6-685A) with human O104:H7 strains (TIF) [file pone.0196490.s003.tif]
